# Comprehensive Analysis of Cardiac Xeno-Graft Unveils Rejection Mechanisms

**DOI:** 10.3390/ijms22020751

**Published:** 2021-01-13

**Authors:** Min Young Park, Bala Murali Krishna Vasamsetti, Wan Seop Kim, Hee Jung Kang, Do-Young Kim, Byeonghwi Lim, Kahee Cho, Jun Seok Kim, Hyun Keun Chee, Jung Hwan Park, Hyun Suk Yang, Harikrishna Reddy Rallabandi, Sun A. Ock, Mi-Ryung Park, Heasun Lee, In-Sul Hwang, Jun-Mo Kim, Keon Bong Oh, Ik Jin Yun

**Affiliations:** 1 Department of Animal Science and Technology, College of Biotechnology and Natural Resources, Chung-Ang University, Gyeonggi-do 17546, Korea; minypark@cau.ac.kr (M.Y.P.); dodamx2@cau.ac.kr (D.-Y.K.); hwi1208@cau.ac.kr (B.L.); 2 Animal Biotechnology Division, National Institute of Animal Science, RDA, Jeollabukdo 55365, Korea; vbmk84@korea.kr (B.M.K.V.); reddy2@korea.kr (H.R.R.); ocksa@korea.kr (S.A.O.); mrpark45@korea.kr (M.-R.P.); leehs1498@korea.kr (H.L.); insuri2642@korea.kr (I.-S.H.); 3 Department of Pathology, Konkuk University Medical Center, Konkuk University School of Medicine, Seoul 05030, Korea; 20050034@kuh.ac.kr; 4 Department of Laboratory Medicine, Hallym University College of Medicine, Hallym University Sacred Heart Hospital, Dongan-gu, Anyang 14068, Korea; kangheejung@hallym.ac.kr; 5 Primate Organ Transplantation Centre, Genia Inc., Sungnam 13201, Korea; kahee.cho@genia.co.kr; 6 Department of Thoracic and Cardiovascular Surgery, Konkuk University Medical Center, Konkuk University School of Medicine, Seoul 05030, Korea; drheart@kuh.ac.kr (J.S.K.); 20050711@kuh.ac.kr (H.K.C.); 7 Department of Nephrology, Konkuk University Medical Center, Konkuk University School of Medicine, Seoul 05030, Korea; pjh@kuh.ac.kr; 8 Department of Cardiology, Konkuk University Medical Center, Konkuk University School of Medicine, Seoul 05030, Korea; yang.hyun@kuh.ac.kr; 9 Department of Surgery, Konkuk University Medical Center, Konkuk University School of Medicine, Seoul 05030, Korea

**Keywords:** porcine cardiac tissue, xenotransplantation, heart failure, transcriptome analysis (or RNA-seq analysis)

## Abstract

Porcine heart xenotransplantation is a potential treatment for patients with end-stage heart failure. To understand molecular mechanisms of graft rejection after heart transplantation, we transplanted a 31-day-old *alpha-1,3-galactosyltransferase knockout* (GTKO) porcine heart to a five-year-old cynomolgus monkey. Histological and transcriptome analyses were conducted on xenografted cardiac tissue at rejection (nine days after transplantation). The recipient monkey’s blood parameters were analyzed on days −7, −3, 1, 4, and 7. Validation was conducted by quantitative real-time PCR (qPCR) with selected genes. A non-transplanted GTKO porcine heart from an age-matched litter was used as a control. The recipient monkey showed systemic inflammatory responses, and the rejected cardiac graft indicated myocardial infarction and cardiac fibrosis. The transplanted heart exhibited a total of 3748 differentially expressed genes compared to the non-transplanted heart transcriptome, with 2443 upregulated and 1305 downregulated genes. Key biological pathways involved at the terminal stage of graft rejection were cardiomyopathies, extracellular interactions, and ion channel activities. The results of qPCR evaluation were in agreement with the transcriptome data. Transcriptome analysis of porcine cardiac tissue at graft rejection reveals dysregulation of the key molecules and signaling pathways, which play relevant roles on structural and functional integrities of the heart.

## 1. Introduction

Heart transplantation is the ultimate therapy for end-stage heart failure patients. However, understanding the molecular mechanisms underlying transplanted graft rejection is challenging. Graft rejection is typically classified as hyperacute rejection, antibody-mediated rejection, acute cellular rejection, or chronic rejection, according to histopathological peculiarities. Histological diagnosis of endomyocardial biopsy based on the guidelines set by the International Society for Heart and Lung Transplantation has been considered the gold standard to detect graft rejection. Accumulated gene expression profiles from histological data allows for the following emerging molecular diagnostics: a microarray-based tool developed to detect acute cellular rejection and antibody-mediated rejection in heart transplants (Molecular Microscope^®^ Diagnostic System, or MMDx) and unsupervised machine learning based on rejection-associated transcripts [1]. Both techniques have been designed for the monitoring of graft rejections [2]. However, none of these approaches have proven sufficiently reliable in enhancing our understanding of the complexity of rejection mechanisms.

Xenografts have been considered as a potential alternative to allografts. Particularly, genetically modified pig heart xenotransplantation (XTx) has become a promising solution due to the adequate size and compatibility of the xenografts as well an increased survival rate due to recent advances in surgical techniques [3,4]. Yet, failures in XTx (e.g., short survival) have frequently occurred due to complex graft rejection mechanisms classified similarly to allograft rejection criteria, such as hyperacute rejection, acute humoral xenograft rejection, acute cellular xenograft rejection, or chronic rejection and vasculopathy [5,6]. Recently, RNA sequencing techniques have been employed successfully for a comprehensive analysis of the global transcriptome and, thus, the molecular mechanisms of dysfunctional cardiac tissue in patients [7,8]. Therefore, the aims of this study were to report the molecular responses of graft rejection at the transcriptome level and to identify the key genes and signaling pathways involved at graft rejection. Pig-to-monkey XTx was used as a model, wherein a genetically modified homozygous *alpha-1,3-galactosyltransferase knockout* (GTKO) pig [9] heart was heterotopically transplanted into a cynomolgus monkey, and RNA sequencing was carried out. We provided information about the key molecules and signaling pathways facilitating the pathophysiological mechanisms in parallel with the standard diagnosis based on hematological and histological analyses of the recipient and grafted donor-heart.

## 2. Results

### 2.1. Recipient Monkey Blood Analysis Showed Systemic Inflammatory Responses at Rejection

Neutrophils, CRP, and fibrinogen are described as indicative markers for innate immunity and their levels are considered as candidate biomolecules at early cardiac rejection from previous studies [10,11,12]. In accordance, our results of hematological and biochemical blood parameters of the recipient monkey during the study period revealed a steady increase in absolute count and percentage of neutrophils accompanied with increased levels of high-sensitivity C-Reactive Protein (hs-crp) and fibrinogen after transplantation, indicating acute inflammatory responses. The level of hemoglobin steadily decreased after transplantation with an increase in red cell distribution width and lactate dehydrogenase level, indicating the development of hemolytic anemia after transplantation. A high level of troponin I on post-op day 7 indicates severe xeno-cardiac damage. Changes in coagulation parameters were not remarkable except for an increase in the level of fibrinogen, reflecting the fact that anticoagulation therapy was not given to the recipient (Table 1).

### 2.2. Histological Assessment of Rejected Xenograft Showed Myocardial Infarction and Fibrosis

**Figure 1:**
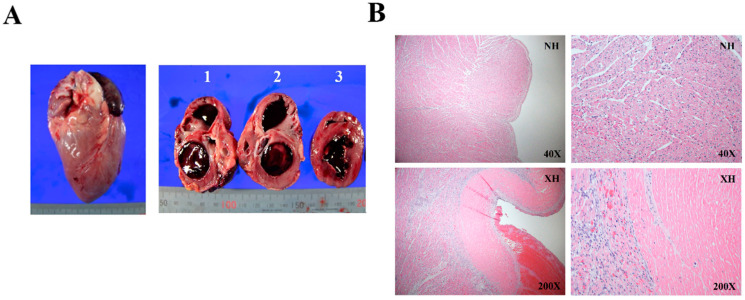
Gross and microscopic findings of the rejected porcine heart (XH). (**A**) Representative images showing anterior (**left**) and interior views (**right**) of XH. Interior views include sagittal plane (1 and 2) and transverse plane (3) of the XH heart. (**B**) Photomicrographs showing endomyocardial infarction and interstitial fibrosis in XH to compare with the normal histological appearance in the control porcine heart(NH).

The rejected porcine heart (XH) exhibited a myocardial pallor, redness, and white fibrosis (Figure 1A). The cardiac chambers were filled with hematoma. Microscopic observation of XH tissue sections showed endomyocardial infarction and patchy interstitial fibrosis (Figure 1B). Intraventricular large organizing hematoma was evident in XH.

### 2.3. Transcriptome Analysis Showed Alterations of a Number of Genes in a Rejected Xenograft 

**Figure 2:**
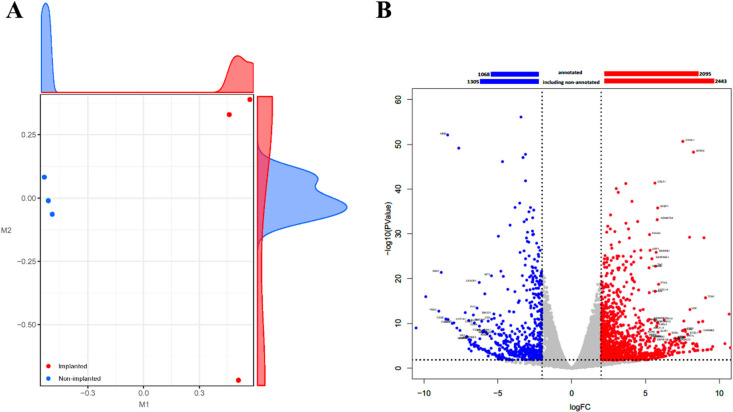
Overview of differentially expressed genes in the rejected porcine heart (XH-DEGs). (**A**) The multi-dimensional scaling plot of total RNAs NH (blue) and XH (red). (**B**) The volcano plot with a bar plot for differentially expressed genes (DEGs) of XH compared to that of NH. Individual colored dots indicate upregulated genes (red, FDR < 0.05 and logFC ≥ 2) and downregulated genes (blue, FDR < 0.05 and logFC ≤ −2) in the treatment group. The labeled genes were the selected top 5% of the upregulated genes annotated by the gene symbol. The bar plot represents the number of DEGs for non-annotated and annotated genes.

The transcriptome analysis of cardiac tissue produced a total of 289 million paired-end sequence reads, with an average of 48 million reads and 51% GC content per sample. The relative expression level of transcripts was normalized to the trimmed means of M (TMM) values and calculated for similarity and dissimilarity between the samples. The multidimensional scaling plot represents the distribution of samples from the rejected porcine heart (XH) and the control porcine heart (NH) (Figure 2A). Samples were closely clustered within the same group, while the samples from XH were clearly distinguished from those from NH. Then, 19,299 porcine genes were narrowed down at FDR < 0.05 and log2 fold change ≥ 2, and 1710 differentially expressed genes (DEGs) were identified between XH and NH. Among them, 2443 genes were upregulated and 1305 genes downregulated in the XH when compared with NH, including 2095 upregulated and 1068 downregulated genes, which are annotated as shown in the volcano plot (Figure 2B).

### 2.4. Functional Annotation and Gene Set Enrichment Analysis (GSEA) Suggested Multiple Molecular Mechanisms Involved in Graft Rejection

**Figure 3:**
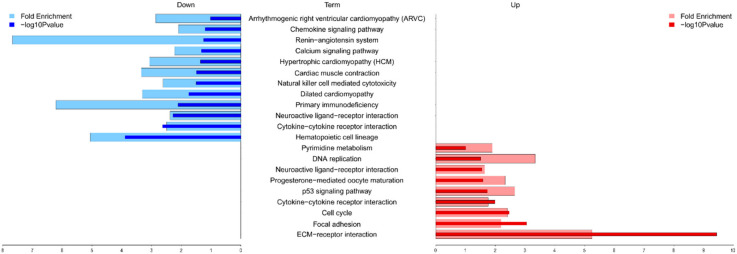
KEGG pathway enrichment analysis of XH-DEGs. Enrichment analysis of pathways associated with XH-DEGs was based on the KEGG databases using the DAVID database (https://david-d.ncifcrf.gov). Down-regulated and up-regulated pathways are shown on the left and right, respectively. The light color represents fold enrichment and dark color indicates −log10 *p*-value. Cutoff *p*-value < 0.05.

**Figure 4:**
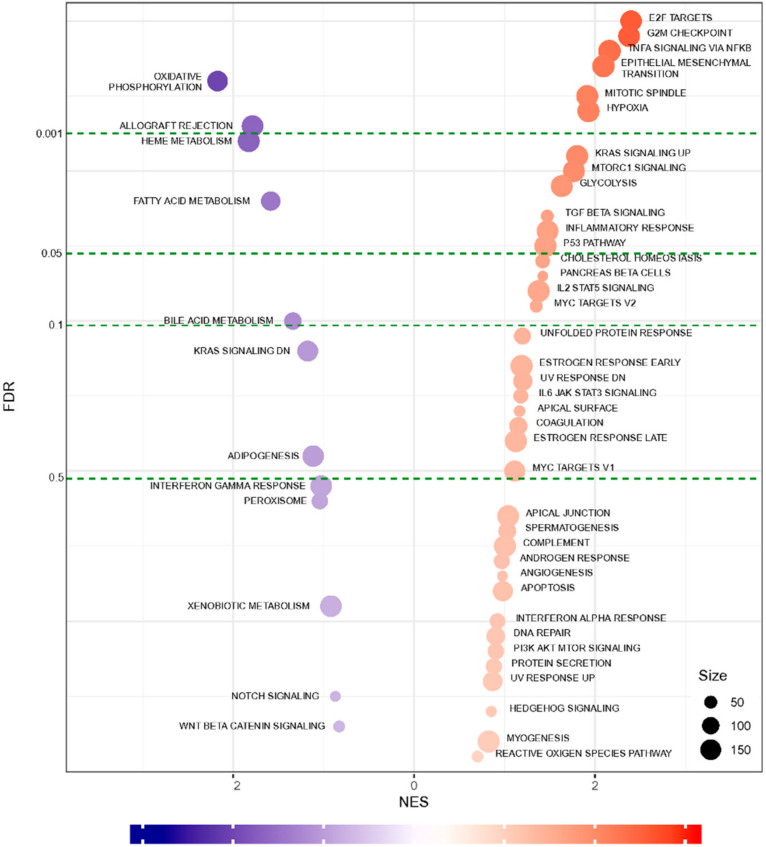
GSEA of whole transcriptome comparisons. A bubble chart of the GSEA hallmark indicates the normalized enrichment scores (NES, *x*-axis) and false discovery rate (FDR, *y*-axis). The size of each circle represents the weighted numbers of genes involved in the term.

To determine the graft rejection-related biological pathways, we performed a series of bioinformatics analyses using DEG data. The Kyoto Encyclopedia of Genes and Genomes (KEGG) pathway enrichment analysis of DEG data revealed 21 pathways that were significantly enriched (Figure 3, Figure S1). Notably, downregulated DEGs were enriched in the pathways associated with a cardiac function, such as hypertrophic cardiomyopathy (HCM) and dilated cardiomyopathy (DCM) as well as signaling pathways (such as calcium and chemokine signaling) (Figure 3 and Table 2). These pathways represent a series of interactions during rejection after heart transplantation in XH. Next, to functionally classify the genes at rejection, we performed GO enrichment analysis of significant DEGs. Table 3 shows significantly clustered functional terms for maintaining intracellular ions of a steady-state through ion transport between cells. Finally, we also performed GSEA analysis for further validation and functional enrichment of gene sets. Figure 4 shows the results obtained using the hallmark database, showing the gene sets that are either up-regulated or down-regulated in XH compared to NH with a normalized enrichment score on the *x*-axis and false discovery rate (FDR) value on the *y*-axis. In the top 10 gene sets, allograft rejection pathways were significantly suppressed, while genes relevant to the cell cycle and inflammatory response pathways were upregulated.

### 2.5. qPCR Validation of Randomly Selected DEGs Confirmed a Similar Expression Trend

We analyzed the transcript level of randomly selected DEGs that presented up-regulation or down-regulation of the calcium signaling pathway (*HTR2C*, *SLC8A3*, *BDKRB1*, *ATP2A2*, and *ADRB1*), CM pathways (*IGF1*, *ADRB1*, and *ACTC1*), cytokine-cytokine receptor interaction (*IL6*), extracellular matrix (ECM) receptor interaction (*ITGA8*, and *TNC*), focal adhesion (*COL1A1*, and *TNC*), cardiac fibroblast markers (*ACTA2*, *COL1A1*, and *VIM*), and cardiac hypertrophy markers (*NPPA*, and *NPPB*) using qPCR. 

**Figure 5:**
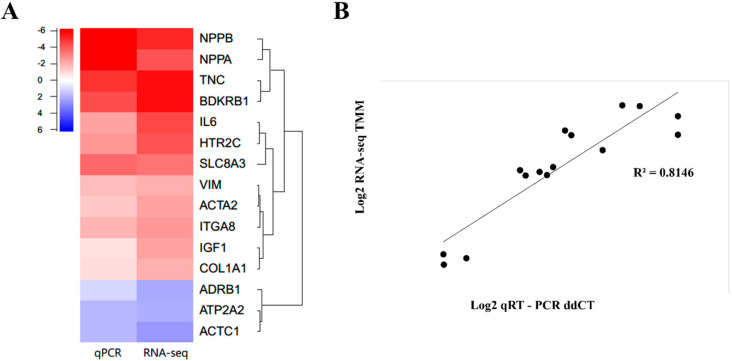
Validation of DEGs by qPCR. DEGs of XH across various biological pathways were verified using qRT-PCR. (**A**) Heat map showing the comparison of expression levels of qPCR vs. RNA sequencing. (**B**) Correlation analysis between XH and NH.

All the quantified genes showed a similar expression trend in qPCR as that observed in RNA sequencing (Figure 5A). The genes depicted in a red color indicate upregulated genes in XH compared to NH, including *Nppb*, *Nppa*, *Tnc*, *Bdkrb1*, *IL6*, *Hrt2c*, *Slc8a3*, *VIM*, *Actin Alpha 2* (*Acta2*), *Integrin Subunit Alpha 8* (*Itga8*), *Igf1*, and *Col1a1*. Similarly, the genes indicated in blue (*Adrb1*, *Atp2a2*, and *Actc1*) were downregulated in XH when it was compared to NH. The intensity of each color represents the degree of the relative expression level. Additionally, relative expression values obtained from qPCR and RNA-seq analysis were significantly correlated (R^2^ = 0.8146) (Figure 5B).

## 3. Discussion

Given that graft rejection is a highly complicated process, a comprehensive understanding of the molecular and/or biochemical mechanisms involved in the initiation and progression of the rejection is lacking. In the present study, as an initial step, we applied next-generation RNA sequencing technology, which is known as a powerful tool for comprehensive analysis of the global transcriptome [7,13,14] to translate and understand rejection mechanisms at a molecular level in early failed porcine XH. 

The functioning of various biological pathways, which are crucial for the maintenance of structural integrity and functional homeostasis of the heart, appears to have been disturbed in XH. The KEGG enrichment analyses of DEGs authentically produced 21 biological pathways, which could be broadly categorized as intracellular signaling pathways, immune functions, and cardiomyopathies (CM) (Figure 3). The downregulated patterns of HCM, DCM, arrhythmogenic right ventricular cardiomyopathy (ARVC), and calcium signaling pathways as well as the upregulated patterns of ECM-receptor interaction pathways observed in this study have been well-known to occur in allograft rejection [15], cardiovascular disease (CVD) [8], and heart failure (HF) [8,13,15], suggesting that the DEG data obtained in this study not only defines the graft rejection mechanisms but is also useful for understanding CVD and HF.

Myocardial infarction and fibrosis observed in XH and the hemolytic anemia revealed in blood parameter analysis suggest the development of microvascular thrombosis in XH. Consistent with these findings, KEGG analysis of DEGs showed an upregulation of ECM-receptor interaction pathways in XH. ECM-receptor interactions are known to be involved in the signaling events that regulate cell survival, growth, shape, differentiation, migration, and motility [16], specifically required in remodeling of the heart [17]. The genes coding for type IA1 collagen (*COL1A1*) and type VIA3 collagen (*CLA6A3*) were increased in XH, which are comparable to animal models of myocardial infarction [18]. Upregulation of type XIA2 collagen (*COL11A2*) in XH corroborates the results of the earlier RNA sequencing study on patients with ischemic cardiomyopathy after allotransplantation [19]. 

We found that the expression of Thrombospondin 1 and 4 (*THBS1* and *4*, or *TSP-1* and *TSP-4*), and Tenascin C (*TNC*) among members of a matricellular proteins (MPs) class were upregulated in XH (Figure S2). MPs, which functionally and structurally interact with extracellular matrix (ECM), and cell surface receptor and signaling molecules, exert fibrogenic actions on cardiomyocytes, fibroblasts, as well as immune and vascular cells in the remodeling of myocardium [18,20]. Members of MPs show different patterns of spatial and temporal upregulation during cardiac remodeling, such as TSP-1 in myocardial infarction, ventricular hypertrophy, and cardiac dilation, TSP-4 in ventricular hypertrophy and myocardial injury, and Tenascin C in tissue injury and fibrosis [21,22,23,24,25,26,27]. Dramatic upregulation of molecules of MPs and ECM in XH appear to lead to prolonged and/or abnormal mechano-transduction processes in myocytes, consequently resulting in HF [28,29].

CM is a well-known heart disease [30], which causes the heart muscle to become enlarged, thick, or rigid (American Heart Association (AHA), www.heart.org) and frequently leads to heart failures in humans [31]. According to AHA, CM can be inherited and acquired, meaning that it can be developed by perturbation of the expression of genes in the heart by the physiological and mechanical stresses of transplantation. The main types of CM by classification based on structural and functional changes [32], such as HCM and DCM, are among the top 10 enriched pathways in XH in this study, indicating that various forms of CM were enforced in XH.

Among the enriched pathways of XH, the calcium signaling pathway showed a pattern of down-regulation, suggesting perturbation of Ca^2+^ homeostasis, which is a major contributor to HF [33]. The expression of multiple calcium handling proteins of cardiomyocytes such as G-protein coupled receptors (*CCKAR, DRD1, PTGER3, CYSLTR2, ADRB1, ADRA1A, HTR2C*, and *HTR2A*) and ion channels (*CACNA1I, CACNA1B, ATP2B3,* and *SLC8A3*) was altered in XH. G-protein coupled receptors are expressed in major cardiac cells and play a central role in normal cardiac health and diseases [34]. Decreased expression of adrenoreceptor beta1 (*ADRB1*) and adrenoreceptor alpha 1A (*ADRA1A*) in XH may contribute to impaired β-adrenoreceptor signaling, a hallmark of HF in humans [35,36]. Ion channels are critical for excitation-contraction coupling of the heart [37] and are often dysregulated in CVD and HF [38]. In addition, our data indicate the accumulation of systemic inflammatory responses in the recipient monkey, which might lead to the destruction of cardiomyocytes in XT with the development of microvascular thrombosis and subsequent infarction. Taken together, we suggest that XH in this study was responding to the pathological tissue remodeling processes comprised of overlapped myocardial infarction, fibrosis, and/or hypertrophy.

Overall, our multi-method study uncovered the critical mechanisms involved in early-day rejected cardiac transplants, like dysregulation of calcium signaling pathway and the relevant factors driving cardiac remodeling processes. Additionally, we unveiled a detailed list of DEGs involved and/or related to the pathological and biochemical outcomes of cardiac transplantation as well as CVDs (Supplementary Table S2). Particularly, the major pathways involved in cardiac graft injection includes immune response (chemokine signaling pathway, cytokine-cytokine signaling pathway, and cytotoxicity), cell cycle/apoptosis (DNA replication, p53 signaling pathway), and structural organization and maintenance (cardiac muscle contraction, calcium signaling pathway) (Figure 3 and Figure 4). Key genes will comprise the major pathways listed above, which are associated with cell-cell interaction and innate immunity (Table 2). Our data are partly consistent with human clinical studies as the major pathways. The related genes discussed in this study were also described in a number of previous studies. Upregulated expression of inflammatory markers in our data are in accordance with previous studies [39,40]. The significance of the genes in the cell cycle can be inferred from the study from Bodez et al., where cardiac allograft recipients received a cell cycle inhibitor in addition to an immunosuppressant [41,42]. The calcium-mediated pathway has been emphasized in Tarazón et al., suggesting sarcoplasmic reticulum Ca^2+^-ATPase (SERCA2a) as a potential non-invasive biomarker of cardiac allograft rejection [43] as well as in Dhar et al. [44]. Although the etiology of cardiac xenograft rejection in this study is equivocal, the comprehensive analysis presents that the discrepancy between the grafted heart and immune system of the recipient as well as physiological damage of the graft resulted in HF with a chronic rejection condition. We believe that the large amount of DEG data obtained in this study could not only provide a crucial clue to complete drawing of functional network topology for progression and manifestation among spatiotemporal molecular mechanisms and pathways related to cardiac rejection and diseases, but also information to develop an advanced molecular diagnostic platform for an accurate assessment of cardiac diseases.

## 4. Methods

### 4.1. Ethics Statements

The experimental procedures were approved by the Orient Bio Institutional Animal Care and Use Committee (IACUC No. ORIENT-IACUC-15053).

### 4.2. Experimental Animals and Heterotopic Transplantation Procedure

A 31-day-old male homozygous *GTKO* pig (generation for which were inbred descendants between cloned heterozygous *GTKO* Chicago mini male pig and wild-type Landrace female pig) was used as a donor (National Institute of Animals Science, Jeollabuk-do, Korea). Weight of porcine heart was 55.0 g at the time of transplantation. The recipient, which is a five-year-old male cynomolgus monkey, was housed in a clean, pathogen-free facility at Genia Inc. (Seongnam, Korea). The bodyweight of the monkey on the day of transplantation was 6.1 kg. Surgical procedures were performed under inhalation anesthesia, which was maintained with 3% of isoflurane (Forane solution^®^, JW phamaceuticals, Seoul, Korea) after intramuscular injection of Ketamine (10 mg/kg) (Ketamine 50^®^, Yuhan corporation, Seoul, Korea). Heterotopic abdominal XTx was performed as described previously [45]. The ascending aorta and root of the donor pig was briefly anastomosed to the recipient monkey’s abdominal aorta and the pig’s main pulmonary artery to the monkey’s inferior vena cava. The pig’s coronary arteries were perfused from the abdominal aorta, and the coronary venous blood entered the right heart via the coronary sinus and was ejected into the inferior vena cava via the pulmonary trunk.

### 4.3. Immunosuppressive Regimen

Immunosuppression was induced in the recipient cynomolgus monkey through treatment with Thymoglobulin (Genzyme, 5 mg·kg^−1^ on days −3, −2, −1, and 0), anti-CD20 antibody (rituximab, Genetech, 20 mg·kg^−1^ on days −7 and 0), and anti-CD154 (NIH Nonhuman Primate Reagent Resource, 20 mg·kg^−1^·day^−1^ ×7, 5C8) for the suppression of T-cell and B-cell activation. Cobra venom factor (Quidel, 0.05 mg·kg^−1^ on days −1, 0, 1, 2, and 3) was used to inhibit complement activation. For maintenance therapy, we applied FK506 (P.O. at 4 mg·kg^−1^·day^−1^), mycophenolate mofetil (P.O. at 100 mg·kg^−1^·day^−1^), and methylprednisolone (I.V. at 1 mg·kg^−1^·day^−1^ for 2 days, tapered down).4.4. Collection of Porcine Cardiac Tissue 

The rejected porcine heart (XH) on post-operation day (POD) 9 was dissected from the cynomolgus monkey in a surgical facility at Genia Inc. Small fragments collected randomly with six samples of ventricles were plunged into liquid nitrogen for RNA sequencing or into 4% paraformaldehyde for histological analysis. An age-matched litter of the donor was sacrificed at NIAS, and samples (NH) were collected.

### 4.4. Hematological and Biochemical Analysis of a Cynomolgus Monkey 

The recipient monkey’s blood parameters were analyzed on days −7, −3, 1, 4, and 7, as described in the Supplementary Materials and methods in detail. 

### 4.5. Histological Analysis

Specimens were fixed in 10% neutral buffered formalin and embedded in paraffin. The paraffin blocks were sliced at thicknesses of 3 µm. The sections were processed by deparaffinization and rehydration and stained with hematoxylin and eosin. 

### 4.6. RNA Sequencing Preparation

One microgram of total RNA prepared by pooling with equal amounts from each sample was used to construct cDNA libraries with the TruSeq RNA library kit. The protocol consisted of polyA-selected RNA extraction, RNA fragmentation, random hexamer-based reverse transcription, and 100 bp paired-end sequencing by Illumina HiSeq 2000 (San Diego, CA, USA). The libraries were quantified using quantitative real-time PCR (qPCR), according to the qPCR Quantification Protocol Guide and qualified using Agilent Technologies 2100 Bioanalyzer (Santa Clara, CA, USA).

### 4.7. Transcriptome Alignment and Differentially Expressed Genes (DEGs) Analysis of the Porcine Cardiac Tissue

A total of 570 million raw RNA sequence reads were produced at an average of 48.2 million sequence reads per sample. Raw sequence reads were quality checked by FastQC [46]. Using the Trimmomatic v0.38 tool, reads were trimmed to remove the adapter sequence, and reads that were below 75 bp were dropped [47]. Trimmed reads were aligned against the swine reference genome (Sus scrofa.Sscrofa11.1.93, GCA_000003025.6) obtained from the Ensembl genome browser (ftp://ftp.ensembl.org/pub/release-95/fasta/sus_scrofa/dna/) by the HISAT2 v2.0.4 tool using default options [48]. Subsequently, the number of reads for each gene was calculated by using Feature Counts v1.6.2 [49]. The relative gene expression level was obtained as the trimmed means of M values (TMM) [50] using an R package edgeR. DEGs were selected with a cut-off of absolute log2 fold change ≥ 2 and a q-value of 0.05 by comparing XH versus NH. The DEGs obtained were used for further analyses. 

### 4.8. Gene Ontology and Pathway Enrichment Analysis

DEGs with Ensembl gene IDs were converted to match human gene IDs and gene symbols using the Database for Annotation, Visualization, and Integrated Discovery (DAVID, https://david.ncifcrf.gov/) [51]. The DEGs were analyzed for functional clustering and enrichment of Gene Ontology (GO) [52] terms and pathways in a biological process, cellular component, molecular function, and Kyoto Encyclopedia of Genes and Genomes (KEGG) pathways using the DAVID program [53]. The most significant enrichment pathway was expressed by a log2 fold enrichment and a −log10 *p*-value. Significant functional annotation clustering was implemented by comparing each enriched GO term with Kappa similarity overlaps and a threshold greater than 3 and 0.5, respectively.

### 4.9. Gene Set Enrichment Analysis (GSEA)

Gene expression data normalized to TMM values were analyzed by the GSEA using the enrichment hallmark database and reactome pathways [54]. The statistical significance (nominal *p*-value) of the enrichment score (ES) was estimated by using an empirical 1000 gene set permutation test procedure that preserves the complex correlation structure of the gene expression data. GSEA results were visualized by a bar plot and the Enrichment Map tool in Cytoscape (*p*-value < 0.005, False discovery rate, FDR < 0.005, and similarity cut off of overlap coefficient ≥ 0.5) [55].

### 4.10. Quantitative Real-Time PCR (qPCR)

Total RNA was extracted from cardiac tissue using RNeasy Mini kit (Qiagen, Hilden, Germany) and cDNA was synthesized using SuperScript IV first-strand cDNA synthesis kit (Invitrogen, Carlsbad, CA, USA). Quantitative real-time PCR was performed using StepOne Real-Time PCR system (ABI, Foster City, CA, USA) and Power SYBR Green PCR Master Mix (ABI). The list of primers used for qPCR is presented in Supplementary Table S1.

### 4.11. Statistical Analysis

All analyses were carried out using the statistic Prism software (GraphPad). One-way ANOVA and Tukey’s post hoc *t*-test was used for statistical analyses. Data are presented as mean ± SE.

## Figures and Tables

**Table 1:** Hematological and biochemical blood parameters of recipient cynomolgus monkey.

Type	Post-Operation Day				
	−7	−3	1	4	7
Calcium (mg/dL)	10.20	10.20	9.00	9.00	9.40
Phosphorus (mg/dL)	6.40	5.10	5.90	5.30	4.50
BUN (mg/dL)	29.00	25.00	32.00	39.00	35.00
Uric acid (mg/dL)	0.30	0.30	<0.10	0.20	0.10
Creatinine (mg/dL)	45.00	1.08	1.09	0.92	0.83
Na (mEq/L)	143.00	147.00	149.00	143.00	142.00
K (mEq/L)	4.90	5.10	5.10	5.30	5.80
Cl (mEq/L)	100.00	106.00	111.00	109.00	103.00
TCO2 (mmol/L)	15.00	19.00	23.00	21.00	23.00
hs-CRP (mg/dL)	0.12	0.20	6.72	1.14	13.68
AST(GOT) (IU/L)	53.00	51.00	1419.00	306.00	103.00
ALT(GPT) (IU/L)	85.00	79.00	255.00	157.00	94.00
Total protein (g/dL)	6.90	7.30	6.10	5.60	5.70
Albumin (g/dL)	3.90	4.20	3.30	3.00	2.80
TBIL (mg/dL)	0.40	0.40	0.40	0.40	0.30
Alk. Phos (IU/L)	513.00	466.00	400.00	332.00	317.00
Chol (mg/dL)	114.00	124.00	103.00	109.00	112.00
GGT (IU/L)	45.00	51.00	40.00	35.00	46.00
LD(LDH) (IU/L)					1997.00
Troponin (ng/mL)					19.10
Tacrolimus (ng/mL)		<2.00	3.70	11.30	
WBC (×10^3^ cells/uL)	10.54	9.63	11.34	10.70	12.97
RBC (×10^7^ cells/uL)	5.18	5.00	4.33	4.10	3.82
Hb (g/dL)	12.10	11.80	10.00	9.40	8.90
Hct (%)	40.50	38.90	33.20	32.70	29.30
MCV (fL)	78.20	77.70	76.70	79.80	76.80
MCH (pg)	23.40	23.60	23.10	22.90	23.40
MCHC (g/dL)	29.90	30.40	30.10	28.70	30.50
RDW (%)	14.60	14.20	13.90	15.10	16.50
Platelet (×10^3^ cells/uL)	457.00	488.00	439.00	516.00	258.00
PCT (%)	0.54	0.38	0.33	0.60	0.21
MPV (fL)	11.90	7.80	7.50	11.60	8.00
PDW	15.00			14.10	
Seg. neut. (%)	38.00	85.70	81.00	75.00	79.00
Lymphocyte (%)	54.00	6.20	3.00	9.00	2.00
Monocyte (%)	7.00	6.20	12.00	13.00	16.00
Eosinophil (%)		0.40	1.00		
Basophil (%)	1.00	0.20			
LUC (%)		1.40			
ANC (uL)	4005.00	8245.00	9299.00	8025.00	10,246.00
PT-INR	0.85	0.76	0.83	0.78	0.82
PT (%)	133.00	166.00	139.00	154.00	142.00
PT (sec)	9.10	8.10	8.90	8.40	8.80
aPTT (sec)	20.70	18.90	19.40	18.10	21.30
Fibrinogen (mg/dL)	114.00	241.00	354.00	270.00	314.00
AT III (%)	108.00	128.00	98.00	94.00	99.00
Protein C (%)	151.00	149.00	164.00	169.00	154.00

POD, post-operation day. TCO2, total CO_2_. Hs-CRP, high-sensitivity C-reactive protein. AST, aspartate aminotransferase. ALT, alanine aminotransferase. TBIL, total bilirubin. Alk.Phos, alkaline phosphatase. Chol, Cholesterol. GGT, Gamma-glutamyl transferase. LD, lactate dehydrogenase. WBC, white blood cell. RBC, red blood cell. Hb, hemoglobin. Hct, hematocrit. MCV, mean corpuscular volume. MCH, mean corpuscular hemoglobin. MCHC, mean corpuscular hemoglobin concentration. RDW, red cell distribution width. PCT, plateletcrit. MPV, mean platelet volume. PDW, platelet distribution width. Seg. Neut., segmented neutrophils. LUC, large unstained cells. ANC, absolute neutrophil count. PT-INR, prothrombin time and international normalized ratio. PT, prothrombin. aPTT, activated partial thromboplastin time. AT III, antithrombin III.

**Table 2:** A summary of the KEGG pathway analysis for DEGs.

Term	Category *	Genes	*p* Value	Fold Enrichment
ECM-receptor interaction	Up	IBSP, TNC, ITGA2, ITGA3, CHAD, HMMR, LAMA1, LAMC3, COMP, ITGA8, ITGB6, COL6A3, SV2B, TNN, COL1A1, SV2A, COL11A2, THBS1, SV2C, THBS2, COL11A1, THBS4, FN1, SPP1	1.386 × 10^−8^	3.9161
Cytokine-cytokine receptor interaction	Both	TNFRSF6B, CSF2, IL1R2, IL22RA1, CCL2, TNFRSF12A, CCR1, TNFSF14, FASLG, CXCR3, CX3CL1, TNFSF18, IL11, CXCL10, LIF, TNFRSF11B, IL12RB1, CCL20, CXCR5, CLCF1, CXCR6, XCR1, IL1A, IL6, IL18RAP, FLT3, IL25, INHBB, CCL11, OSM, INHBA, PRLR, CXCL14, CCR3, CCR2, CX3CR1, NGFR, XCL1	5.430 × 10^−5^	1.9879
Hematopoietic cell lineage	Down	CSF2, IL1R2, IL6, CD3G, CD3D, CD8A, FLT3, CD3E, ITGA2, ANPEP, ITGA3, IL11, CD38, DNTT, MS4A1, CD2, CD5, IL1A	1.170 × 10^−4^	2.8687
Neuroactive ligand-receptor interaction	Up	F2RL3, MCHR1, CCKAR, DRD1, GABRB3, GABRB2, CYSLTR2, DRD2, GABRB1, F2RL1, GRIK5, BDKRB1, HCRTR1, P2RY6, PTGIR, HRH3, NMUR1, CNR2, GABRD, PTGER3, GABRA1, GABRA4, GRIN1, GRIN2A, NTSR2, GRM4, P2RY10, CHRM4, ADRB1, PRLR, MLNR, ADRA1A, FSHB, HTR2C, HTR2A	3.664 × 10^−4^	1.8739
Focal adhesion	Up	IBSP, PGF, TNC, CHAD, COMP, ITGB6, COL6A3, TNN, THBS1, COL11A2, COL11A1, THBS2, THBS4, FN1, SPP1, MYLK3, ITGA2, IGF1, ITGA3, MAPK10, LAMA1, RASGRF1, LAMC3, ITGA8, COL1A1	1.030 × 10^−2^	1.7048
Hypertrophic cardiomyopathy	Down	ACE, ACTC1, IL6, ATP2A2, ITGA8, CACNG6, ITGB6, CACNG5, CACNB2, IGF1, ITGA2, ITGA3	4.275 × 10^−2^	1.935
Calcium signaling pathway	Down	SLC8A3, CCKAR, DRD1, PTGER3, ERBB4, CYSLTR2, MYLK3, GRIN1, CACNA1I, GRIN2A, BDKRB1, CD38, GNAL, ATP2B3, ADRB1, ATP2A2, ADRA1A, HTR2C, HTR2A, CACNA1B	5.215 × 10^−2^	1.5575
Cell cycle	Up	CDC6, PKMYT1, ESPL1, MCM2, MCM3, MCM4, MCM5, CCNB1, CDKN1A, CCNB3, CCNB2, PLK1, BUB1, BUB1B, MYC	6.859 × 10^−2^	1.6447
Dilated cardiomyopathy	Down	ACTC1, ADRB1, ATP2A2, ITGA8, CACNG6, ADCY6, ITGB6, CACNG5, CACNB2, IGF1, ITGA2, ITGA3	6.876 × 10^−2^	1.7878
Bladder cancer	Both	RPS6KA5, CDKN1A, PGF, MMP9, CDH1, THBS1, MYC	8.108 × 10^−2^	2.2844
Progesterone-mediated oocyte maturation	Up	CCNB1, CCNB3, CCNB2, PLK1, ADCY6, BUB1, PKMYT1, IGF1, MAPK11, MAPK10, CPEB1	9.290 × 10^−2^	1.7531

* Category indicates KEGG results based on gene regulation profiles of up, down, and both, respectively.

**Table 3:** Top five enriched functional gene ontology (GO) of DEGs.

Cluster	Enrichment Score	Category *	Term	Count	*p*-Value	Fold Enrichment
1	1.652	CC	cell-cell adherens junction	25	5.00 × 10^−3^	1.834
		MF	cadherin binding involved in cell-cell adhesion	20	4.00 × 10^−2^	1.61
		BP	cell-cell adhesion	19	5.00 × 10^−2^	1.622
2	1.522	MF	voltage-gated potassium channel activity	8	1.00 × 10^−2^	3.22
		BP	potassium ion transmembrane transport	12	2.00 × 10^−2^	2.294
		MF	delayed rectifier potassium channel activity	6	2.00 × 10^−2^	3.891
		CC	voltage-gated potassium channel complex	9	3.00 × 10^−2^	2.424
		BP	regulation of ion transmembrane transport	10	5.00 × 10^−2^	2.084
		BP	potassium ion transport	7	1.00 × 10^−1^	1.974
3	1.413	BP	membrane repolarization during cardiac muscle cell action potential	4	8.00 × 10^−3^	9.252
		BP	regulation of membrane repolarization	4	1.00 × 10^−2^	7.71
		MF	voltage-gated potassium channel activity involved in cardiac muscle cell action potential repolarization	3	3.00 × 10^−2^	10.007
		BP	positive regulation of potassium ion transmembrane transport	3	1.00 × 10^−1^	4.626
		BP	regulation of heart rate by cardiac conduction	4	2.00 × 10^−1^	2.643
4	1.373	CC	spectrin	5	3.00 × 10^−4^	13.166
		BP	actin filament capping	4	2.00 × 10^−2^	7.117
		CC	spectrin-associated cytoskeleton	3	4.00 × 10^−2^	8.887
		MF	structural constituent of cytoskeleton	9	1.00 × 10^−1^	1.91
		MF	phospholipid binding	6	3.00 × 10^−1^	1.629
		BP	ER to Golgi vesicle-mediated transport	6	8.00 × 10^−1^	0.867
5	1.333	MF	glutathione peroxidase activity	5	1.00 × 10^−2^	5.559
		BP	response to reactive oxygen species	6	3.00 × 10^−2^	3.558
		BP	cellular oxidant detoxification	5	4.00 × 10^−1^	1.652

* BP, Biological process. CC, Cellular component. MF, Molecular function.

## Data Availability

Data is contained within the article or Supplementary Material.

## Supplementary Materials

The following are available online at https://www.mdpi.com/1422-0067/22/2/751/s1. Figure S1: Network of gene set enrichment analysis (GSEA) using the Reactome database. Figure S2: Immunohistochemistry of matricellular proteins from heart sections of XH and NH. Table S1: List of primers used for qPCR.
