# Supplementary material for: Comprehensive Analysis of Cardiac Xeno-Graft Unveils Rejection Mechanisms
Source: Int J Mol Sci. 2021 Jan 13;22(2):751. doi: 10.3390/ijms22020751 (PMC7828557; doi:10.3390/ijms22020751)
Supplement: Supplementary file 1 [file ijms-22-00751-s001.pdf]

SUPPLEMENTAL MATERIAL

Supplemental materials and methods

Hematological and biochemical analysis of cynomolgus monkey

ADVIA 21020 instrument (Siemens, Munich, Germany) was used for hematological analysis; erythrocytes (RBC), leukocytes (WBC), hemoglobin (Hb), hematocrit (Hct), red blood cell distribution width (RDW), mean corpuscular volume (MCV), mean corpuscular hemoglobin (MCH), mean corpuscular hemoglobin concentration (MCHC), platelets, plateletcrit (PCT), mean platelet volume (MPV), segmented neutrophils (Seg.neut.), absolute neutrophil counts (ANC), monocytes, lymphocytes, basophils, eosinophils, and large unstained cells (LUC).

AU400 instrument (Olympus, Tokyo, Japan) was used for biochemical analysis; total cholesterol (Chol), blood urea nitrogen (BUN), uric acid, total carbon dioxide (TCO2), creatinine, aspartate aminotransferase (AST/GOT), alanine aminotransferase (ALT/GPT), alkaline phosphatase (Alk Phos), gamma-glutamyl transferase (GGT), lactate dehydrogenase (LD), total bilirubin (TBIL), total protein, albumin, high sensitive C-reactive protein (hs-CRP), sodium (Na), potassium (K), chloride (Cl), calcium, and phosphorous .

ACL7000 (Beckman Coulter, California, USA) was used for coagulation relevant parameter analysis; activated partial thromboplastin time (aPTT), prothrombin time (PT, %; sec; and international normalized ratio (INR)), protein C, antithrombin III (AT III), and fibrinogen.

Architect i2000SR (Abbott Laboratories, Illinois, USA) was used for immunoassay of troponin I and tacrolimus.

Supplementary Table 1. List of primers used for qPCR.

Gene	Forward primer	Reverse primer	Sequence ID
NPPA	GGGGTAGGATGGACAGGATT	CAGCAACGAGACAGGACAAA	NM_214260.2
NPPB	GCAGCAGCCTCTATCCTCTC	TCCTGTATCCCTGGCAGTTC	NM_213846.1
TNC	GAGAAGACTGCTCCCAGGTG	CTCCAGGGACTCTGAACTGC	NM_214230.2
BDKRB1	TCTGCCGCGTGGTCAAC	ACCACCAGGAAGATGCTGATG	NM_001113064.1
IL6	GCGCAGCCTTGAGGATTTC	CCCAGCTACATTATCCGAATGG	NM_214399.1
HTR2C	GCAAGAGGAATACGGATGAAGAGA	GGCGCGGGTTCAAATCT	NM_001286590.1
SCL8A3	CTTCCCTTGGAGACAAGATTGC	TGGATACCCCAAGGAACATGTAT	XM_013989127.2
VIM	AAGAGAACTTTGCCGTGGAA	TCCAGCAGCTTCCTGTAGGT	XM_005668106.3
ACTA2	CTCCTGAGCGCAAATACTCC	ACAGCGAGCAGGGTAAGTGT	NM_001164650.1
ITGA8	CGGCTGTAGCTCAAGTGGAAAT	TCCGGTTCCCAGTTGTGAAT	XM_021064834.1
IGF1	ACAAGGTTAAGATGCACATCACATC	GCAAGCACAGGGCCAAGT	NM_214256.1
COL1A1	CCAAGAAGAAGGCCAACAAG	ACGTCATCGCACAACACATT	XM_021067153.1
ADRB1	CCAAGTGCTGCGATTTCGT	TGCACAAGGGCACGTAGAAG	NM_001123074.1
ATP2A2	GTCGCATTGGCATCTTTGG	TCCCGACCTGTAAAAGCCTTT	NM_213865.1
ACTC1	ACTGAGGCCCCCCTGAAC	TTTCAAACATGATCTGGGTCATCT	NM_001170517.2

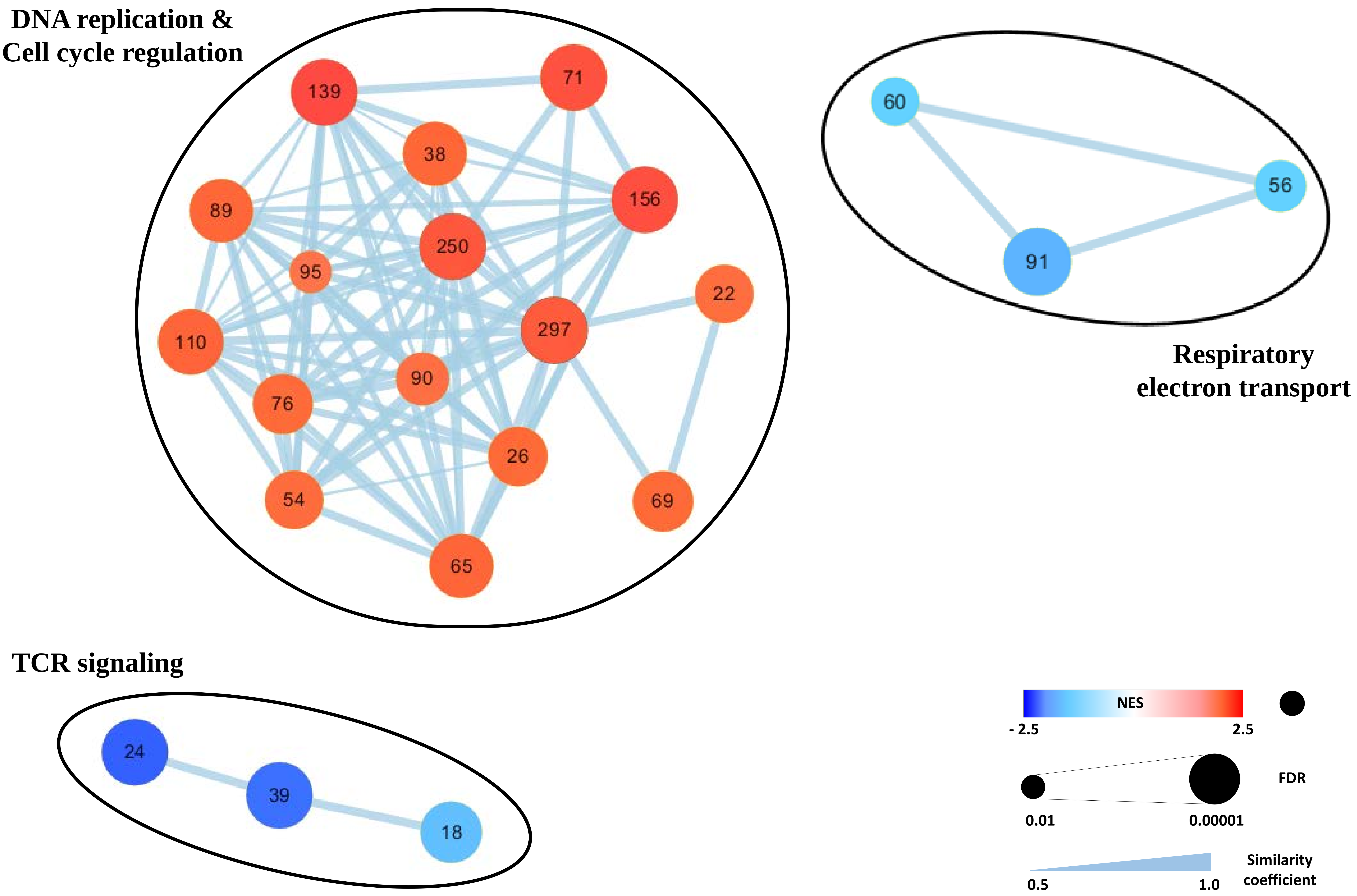

Supplementary figure 1. Network of gene set enrichment analysis using the Reactome database. The network of the whole transcriptome with conservative statistical significance. (*P* - value < 0.005, FDR < 0.005 and overlap coefficient ≥ 0.5). Nodes represent enriched gene sets, where node sizes show FDR, filled insides corresponds to NES and labels based on the number of genes in the gene set after filtering out those genes not in the expression dataset. Edges indicate overlap of gene between set of Reactome pathway through the line thickness correlating with the degree of overlap.

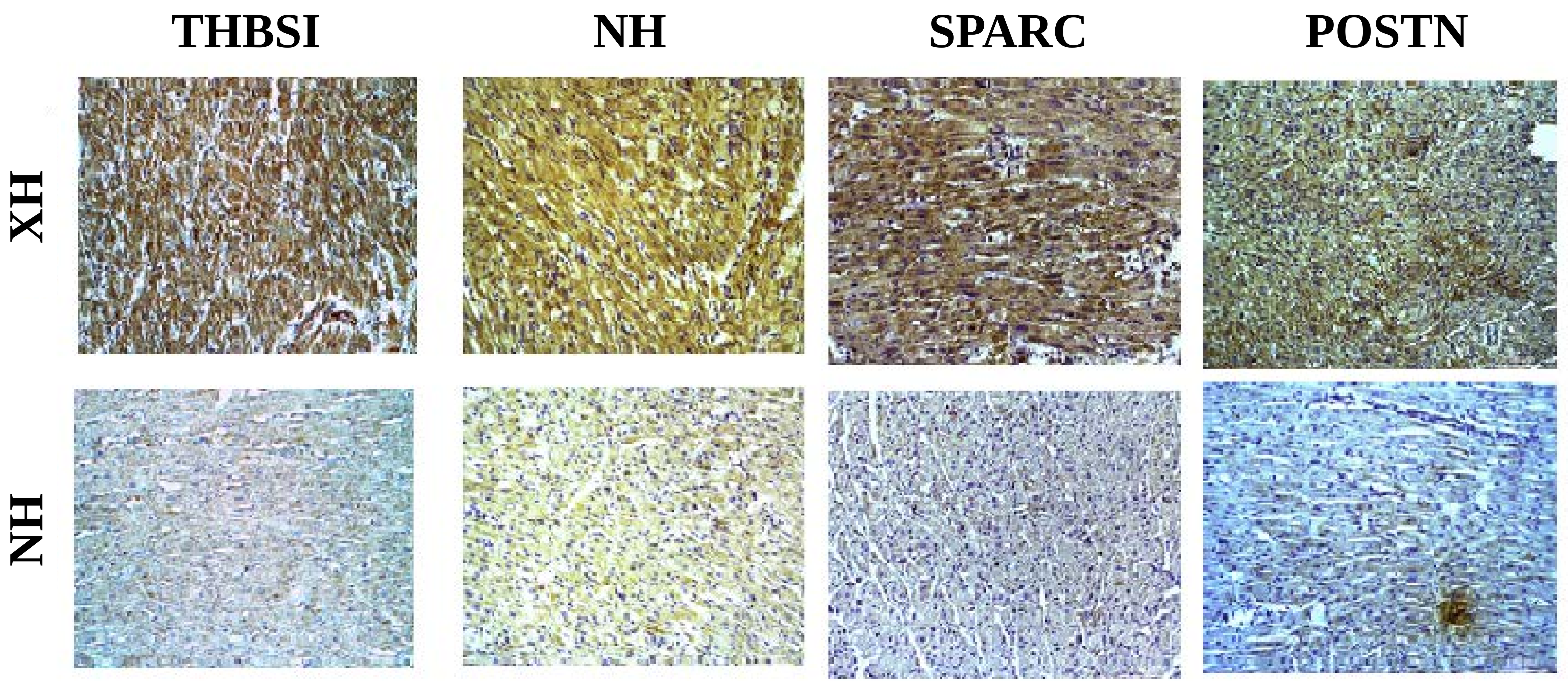

Supplemental figure 2. Immunohistochemistry of matricellular proteins from heart sections of XH and NH. THBS I, Thrombospondin I; TNC, Tenascin C, SPARC, Secreted protein acidic and rich in cysteine; POSTN, Periostin.
